# Kaposi’s Sarcoma-Associated Herpesvirus Lytic Replication Interferes with mTORC1 Regulation of Autophagy and Viral Protein Synthesis

**DOI:** 10.1128/JVI.00854-19

**Published:** 2019-10-15

**Authors:** Eric S. Pringle, Carolyn-Ann Robinson, Craig McCormick

**Affiliations:** a Department of Microbiology and Immunology, Dalhousie University, Halifax, Nova Scotia, Canada; b Beatrice Hunter Cancer Research Institute, Halifax, Nova Scotia, Canada; c Department of Microbiology, Immunology and Infectious Diseases, University of Calgary, Calgary, Alberta, Canada; University of Southern California

**Keywords:** KSHV, autophagy, eIF4F, lytic, mTORC1, translation initiation

## Abstract

Mechanistic target of rapamycin complex 1 (mTORC1) is a master regulator of cellular metabolism. In nutrient-rich environments, mTORC1 kinase activity stimulates protein synthesis to meet cellular anabolic demands. Under nutrient-poor conditions or under stress, mTORC1 is rapidly inhibited, global protein synthesis is arrested, and a cellular catabolic process known as autophagy is activated. Kaposi’s sarcoma-associated herpesvirus (KSHV) encodes multiple proteins that stimulate mTORC1 activity or subvert autophagy, but precise roles for mTORC1 in different stages of KSHV infection remain incompletely understood. Here, we report that during latent and lytic stages of KSHV infection, chemical inhibition of mTORC1 caused eukaryotic initiation factor 4F (eIF4F) disassembly and diminished global protein synthesis, which indicated that mTORC1-mediated control of translation initiation was largely intact. We observed that mTORC1 was required for synthesis of the replication and transcription activator (RTA) lytic switch protein and reactivation from latency, but once early lytic gene expression had begun, mTORC1 was not required for genome replication, late gene expression, or the release of infectious progeny. Moreover, mTORC1 control of autophagy was dysregulated during lytic replication, whereby chemical inhibition of mTORC1 prevented ULK1 phosphorylation but did not affect autophagosome formation or rates of autophagic flux. Together, these findings suggest that mTORC1 is dispensable for viral protein synthesis and viral control of autophagy during lytic infection and that KSHV undermines mTORC1-dependent cellular processes during the lytic cycle to ensure efficient viral replication.

**IMPORTANCE** All viruses require host cell machinery to synthesize viral proteins. A host cell protein complex known as mechanistic target of rapamycin complex 1 (mTORC1) is a master regulator of protein synthesis. Under nutrient-rich conditions, mTORC1 is active and promotes protein synthesis to meet cellular anabolic demands. Under nutrient-poor conditions or under stress, mTORC1 is rapidly inhibited, global protein synthesis is arrested, and a cellular catabolic process known as autophagy is activated. Kaposi’s sarcoma-associated herpesvirus (KSHV) stimulates mTORC1 activity and utilizes host machinery to synthesize viral proteins. However, we discovered that mTORC1 activity was largely dispensable for viral protein synthesis, genome replication, and the release of infectious progeny. Likewise, during lytic replication, mTORC1 was no longer able to control autophagy. These findings suggest that KSHV undermines mTORC1-dependent cellular processes during the lytic cycle to ensure efficient viral replication.

## Abstract (precis)

All viruses require host cell machinery to synthesize viral proteins. A host cell protein complex known as mechanistic target of rapamycin complex 1 (mTORC1) is a master regulator of protein synthesis. Under nutrient-rich conditions, mTORC1 is active and promotes protein synthesis to meet cellular anabolic demands. Under nutrient-poor conditions or under stress, mTORC1 is rapidly inhibited, global protein synthesis is arrested, and a cellular catabolic process known as autophagy is activated. Kaposi’s sarcoma-associated herpesvirus (KSHV) stimulates mTORC1 activity and utilizes host machinery to synthesize viral proteins. However, we discovered that mTORC1 activity was largely dispensable for viral protein synthesis, genome replication, and the release of infectious progeny. Likewise, during lytic replication, mTORC1 was no longer able to control autophagy. These findings suggest that KSHV undermines mTORC1-dependent cellular processes during the lytic cycle to ensure efficient viral replication.

## INTRODUCTION

Kaposi’s sarcoma-associated herpesvirus (KSHV) is the infectious cause of Kaposi’s sarcoma (KS), primary effusion lymphoma (PEL), and multicentric Castleman’s disease (1–3). KSHV is a gammaherpesvirus that establishes lifelong latency in B lymphocytes and readily establishes latency in cultured epithelial and endothelial cells (4), although retention of the episome is unstable (5). Latent gene expression is limited to a discrete subset of viral proteins and microRNAs (miRNAs) that dysregulate the cell cycle, reorganize the cytoskeleton, and stimulate the production of proangiogenic cytokines and growth factors that contribute to tumorigenesis (6–10). Cells expressing KSHV lytic proteins or *de novo* infections also contribute to the proinflammatory and proangiogenic environment of the lesion (10–13). Reactivation requires the expression of the immediate early (IE) lytic switch protein replication and transcriptional activator (RTA) (open reading frame 50 [ORF50]), which initiates an ordered, temporal cascade of gene expression (14, 15).

Mechanistic target of rapamycin complex 1 (mTORC1) activation is a hallmark of KSHV infection *in vitro* and *in vivo* (16–20, 22). mTOR is a serine/threonine kinase that is incorporated into two large protein complexes, mTORC1 and mTORC2, which are assembled by mTOR association with Raptor and Rictor, respectively. mTORC1 is activated by growth signals such as insulin and nutrient abundance. Active mTORC1 phosphorylates target proteins that support translation, suppress autophagy, and promote the synthesis of lipids and nucleic acids. mTORC1 substrate proteins are phosphorylated in KS lesions, and treatment of iatrogenic KS with the allosteric mTORC1 inhibitor rapamycin caused regression of KS tumors (16), likely due to diminished production of the key host angiogenic growth factor vascular endothelial growth factor A (VEGF-A) (17, 21). Inhibition of mTORC1 restricts PEL proliferation *in vivo* and *in vitro* by reducing the production of autocrine growth factors (19, 22). However, despite the clear importance of mTORC1 signaling in KS and PEL, little is known about the role of mTORC1 during lytic replication.

mTORC1 kinase activity can be inhibited by small molecules. The eponymous rapamycin binds to FKBP12 and forms an allosteric inhibitory complex that binds to mTORC1 (23). mTOR active-site inhibitors were subsequently developed that directly inhibited kinase activities of both mTORC1 and mTORC2. Torin-1 (here Torin) was developed by optimization of a lead compound discovered in a small-molecule screen for mTORC1 inhibitors. Torin is a highly specific and potent active-site inhibitor of mTOR with a 50% inhibitory concentration (IC_50_) in the low nanomolar range (24). Torin is more effective than rapamycin in limiting the phosphorylation of mTORC1 targets due to differences in the quality of mTORC1 substrates. Quality is determined by the peptide sequence surrounding the phosphorylation site: low-quality sites are rapidly dephosphorylated during rapamycin treatment, whereas high-quality substrates are dephosphorylated only during starvation or treatment with active-site mTOR inhibitors (25).

Several early lytic KSHV proteins stimulate or mimic mTORC1 activation (reviewed in reference 26). Both the viral B cell receptor homolog known as K1 and the viral CXCR2 chemokine receptor homolog known as viral G protein-coupled receptor (vGPCR) activate mTORC1 by stimulating the upstream mTORC1 kinase Akt (18, 27, 55). The viral serine/threonine kinase ORF36 mimics the mTORC1 substrate ribosomal protein S6 kinase 1 beta (RPS6KB1) (better known as p70S6K1) and phosphorylates a similar array of substrates (28). ORF45 assembles an activated complex of extracellular signal-regulated kinase (ERK) and ribosomal protein S6 kinase A1 (RSK) that stimulates the phosphorylation of eukaryotic initiation factor 4B (eIF4B) and ribosomal protein S6 (S6), which are normally phosphorylated in an mTORC1/p70S6K1-dependent manner (29). ORF45 is also required to support mTORC1 activation in KSHV-infected lymphatic endothelial cells, but the exact signaling pathway is unclear (12). The existence of KSHV mTORC1-activating proteins in the lytic gene expression program suggests a proviral role for mTORC1, but this has not yet been fully examined.

Nutrient withdrawal inactivates mTORC1 and limits translation initiation (30, 31). Herpesvirus mRNAs are transcribed by host RNA polymerase II (Pol II) and posttranscriptionally modified by host 7-methyl guanosine (m^7^G) capping, polyadenylation, and, in some instances, mRNA splicing machinery to prepare them for translation. Capped mRNAs generally access ribosomes through the actions of the eIF4F complex, which comprises the cap-binding protein eIF4E, the RNA helicase eIF4A, and the large scaffolding protein eIF4G1 or the alternative scaffolding protein eIF4G3 (30, 31). Assembly of eIF4F is regulated by eIF4E-binding proteins like 4E-BP1, which prevent the association of eIF4E and eIF4G. Under nutrient-rich conditions that support high levels of protein synthesis, 4E-BP1 is maintained in an inactive form by mTORC1-mediated phosphorylation (32–34). When mTORC1 activity is lost, hypophosphorylated 4E-BP1 binds eIF4E to limit translation initiation. Consistent with the action of mTORC1-promoting activities of K1, vGPCR, and ORF45, 4E-BP1 is hyperphosphorylated, and eIF4F complexes can assemble during KSHV lytic replication (35). This suggests that eIF4F is available for translation of viral and host mRNAs alike during lytic replication. However, the precise roles of mTORC1 and eIF4F in supporting viral and host protein synthesis during the KSHV lytic cycle remain largely unexplored.

mTORC1 is also a key regulator of the bulk cellular catabolic process known as autophagy; in response to nutrients and mitogenic signals, mTORC1 phosphorylates and inactivates ULK1, ULK2, and ATG13, thereby preventing the initiation of autophagy (36). Several viral proteins limit autophagy during lytic replication, but whether mTORC1 activation contributes to this antagonism remains unclear. The lytic viral Bcl-2 homolog (vBcl-2) binds to Beclin1 and prevents autophagosome formation (37), whereas the viral survivin homolog K7/K-survivin binds to Rubicon and interferes with autophagosome maturation by blocking Vps34 kinase activity (38). The latent viral FLICE-inhibitory protein (vFLIP) may also contribute to autophagy suppression during the early lytic cycle by binding to and inhibiting ATG13, an E2-like enzyme involved in microtubule-associated protein 1A/1B light chain 3 (LC3) conjugation to phosphatidylethanolamine, thereby inhibiting the formation of the phagophore (39). In addition to the actions of these proteins, KSHV stimulation of mTORC1 activity during the lytic cycle would normally be expected to prevent the initiation of autophagy. For example, K1 and vGPCR stimulation of Akt kinase activity and mTORC1 phosphorylation should restrain autophagy during the early stages of the lytic cycle, but the precise contribution of mTORC1 regulation to the control of autophagy during KSHV lytic replication remains unclear.

Here, using PEL cell (TREx-BCBL1-RTA) and epithelial cell (iSLK.219) models and the allosteric mTORC1 inhibitor rapamycin or the competitive mTORC1 inhibitor Torin, we examined the requirement of mTORC1 in KSHV reactivation from latency, viral protein synthesis, and virion production. We also examined how KSHV infection impacts the ability of mTORC1 to control autophagy. Despite contributing to global protein synthesis, mTORC1 activity was not required for genome replication, late gene transcription, and the release of infectious progeny. Likewise, KSHV lytic replication subverted normal mTORC1 control of autophagy. Conversely, mTORC1 was required to support the synthesis of the KSHV lytic switch protein RTA and efficient reactivation from latency. These findings suggest that KSHV relies on mTORC1 for lytic reactivation but undermines mTORC1-dependent cellular processes during the lytic cycle to ensure efficient viral replication.

## RESULTS

### mTORC1 is insensitive to amino acid starvation during KSHV lytic infection.

**FIG 1:**
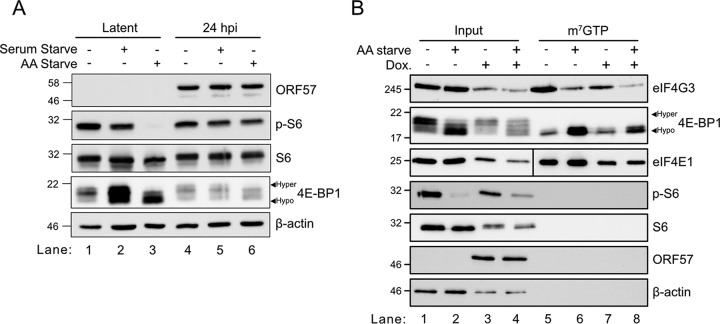
mTORC1 is insensitive to amino acid starvation during KSHV lytic infection. (A) TREx-BCBL1-RTA cells were seeded onto poly-d-lysine-coated dishes and left overnight to adhere. The following day, cells were washed with PBS and incubated in full medium or starvation medium lacking serum or amino acids (AA) for 1 h prior to harvest. mTORC1 activity was determined by immunoblotting cell lysates for phosphorylation of the mTORC1 substrates S6 and 4E-BP1. (B) Same as panel A but with cell lysates harvested under nondenaturing conditions and incubated with m^7^GTP-agarose beads to isolate cap-binding protein complexes. To assess the integrity of eIF4F, cell lysates or m^7^GTP pulldowns were probed for eIF4E1, 4E-BP1, and eIF4G3 by Western blotting. Lytic replication was visualized by probing for the KSHV early protein ORF57.

Amino acid (AA) depletion normally causes mTORC1 inactivation, yet previous studies have shown that herpes simplex virus 1 (HSV-1) and human cytomegalovirus (HCMV) proteins maintain mTORC1 activity in the absence of AAs, which enhances virion production (42, 74). KSHV encodes several proteins that intercede in the mTORC1 signaling pathway (26), but it is not yet known whether KSHV can similarly render mTORC1 insensitive to nutrient depletion. To test this directly, the PEL cell line TREx-BCBL1-RTA, which bears a doxycycline (dox)-regulated RTA transgene, was treated with dox for 24 h to stimulate reactivation from latency or left untreated to maintain latency. After 24 h, the cells were washed with phosphate-buffered saline (PBS) and incubated for 1 h in complete medium, serum-free medium, or AA-free medium supplemented with 10% dialyzed fetal bovine serum (FBS), prior to harvest. There was no change in the phosphorylation of the mTORC1 substrate ribosomal protein S6 (S6) following serum starvation of latently infected or lytically infected cells, compared to controls (Fig. 1A). As expected, after 1 h of AA withdrawal, mTORC1 was completely inactivated in latently infected cells, with no evidence of S6 or 4E-BP1 phosphorylation. In contrast, in lytic infection, AA starvation had little effect on S6 phosphorylation or 4E-BP1 phosphorylation, as determined by slowed migration on SDS-PAGE gels. Lytic replication also reduced total levels of 4E-BP1 protein. Together, these findings indicate that, similar to HSV-1 and CMV infection, lytic KSHV infection maintains mTORC1 activity under AA-deficient conditions that would normally inactivate it.

mTORC1 activity during KSHV replication was previously shown to enable the assembly of the eIF4F cap-binding complex (35). To address the functional relevance of starvation-resistant mTORC1 activity during KSHV lytic infection, latently infected TREx-BCBL1-RTA cells were mock treated or treated with dox to induce lytic reactivation for 24 h prior to 1 h of treatment with full medium or AA-deficient medium, followed by harvesting of lysates under nondenaturing conditions to preserve the integrity of preexisting protein complexes. These lysates were incubated with m^7^GTP-agarose beads to allow eIF4F assembly on artificial cap structures, followed by affinity purification. As described above, we observed that 1 h of AA starvation rapidly shut off S6 phosphorylation in latent cells but not lytic cells (Fig. 1B, lanes 1 to 4). The eIF4F components eIF4E1 and eIF4G3 could be isolated on m^7^GTP-agarose beads from latently infected cells (Fig. 1B, lane 5), and 1 h of AA starvation increased the association of inhibitory hypophosphorylated 4E-BP1 complexes in the pulldowns and decreased the isolation of eIF4G3 (Fig. 1B, lane 6), which indicated that mTORC1 inactivation increased the proportion of hypophosphorylated 4E-BP1 in the lysate, which was then able to inhibit eIF4F assembly on cap structures as expected. In lytically infected cells, even though mTORC1 was largely insensitive to AA withdrawal and a high proportion of the total 4E-BP1 remained hyperphosphorylated (Fig. 1B, lane 4), this was insufficient to prevent 4E-BP1 inhibition of eIF4F assembly in our system (Fig. 1B, lanes 7 and 8). Thus, KSHV lytic infection sustains mTORC1 activity during AA withdrawal, but this is insufficient to prevent eIF4F disassembly.

### mTOR is active during lytic replication but is not required for progression from early to late replication.

**FIG 2:**
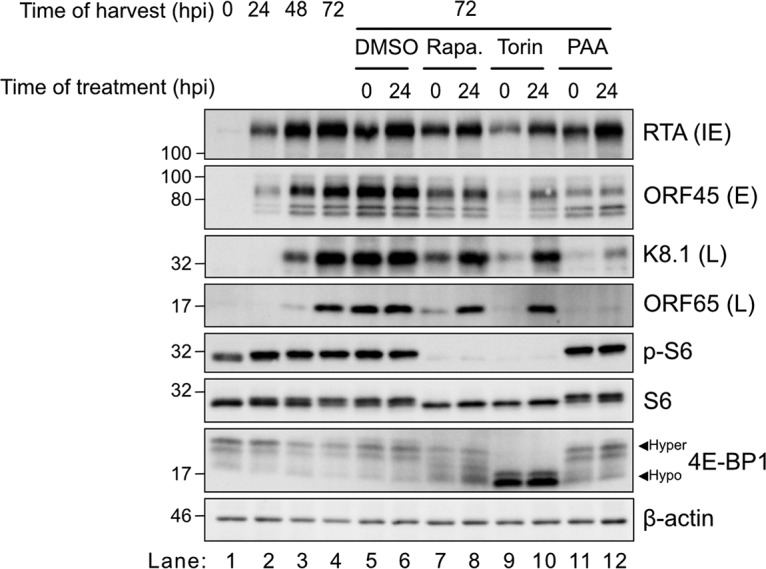
mTOR is active during KSHV lytic replication but is not required for synthesis of late proteins. iSLK.219 cells were treated with 1 μg/ml dox to induce lytic reactivation. The DMSO vehicle control, 250 nM Torin, 250 nM rapamycin, or 500 μM phosphonoacetic acid (PAA) was added at either 0 or 24 hpi. Lysates were harvested at the indicated times and probed for the immediate early protein RTA, the early protein ORF45, and the late proteins K8.1 and ORF65. mTORC1 activity was determined by probing for phospho-S6 and 4E-BP1. The housekeeping protein β-actin was probed as a loading control.

KSHV lytic replication features mTORC1 activation (12, 26, 35), but it is not known whether mTORC1 activity is required for viral protein synthesis and progression through the replication cycle. We tested the requirement for mTORC1 activity in KSHV-infected iSLK.219 epithelial cells; dox addition to iSLK.219 cells stimulates the accumulation of RTA over a 72-h time course (Fig. 2, lanes 1 to 4). The early (E) viral protein ORF45 was readily detected by 24 h postinduction (hpi) (Fig. 2, lane 2), whereas the late (L) proteins K8.1 and ORF65 began to accumulate by 48 hpi (Fig. 2, lane 3). As expected, treatment of cells with the herpesvirus DNA polymerase inhibitor phosphonoacetic acid (PAA) at 0 or 24 hpi prevented late protein accumulation, whereas RTA (IE) and ORF45 (E) accumulation was unaffected (Fig. 2, lanes 11 and 12). Because PAA inhibited late protein accumulation when delivered at 24 hpi, KSHV genome replication must have occurred after 24 hpi in this model. Rapamycin is an allosteric mTORC1 inhibitor that does not target mTORC2 (24, 25, 43). In iSLK.219 cells, rapamycin treatment delivered at the time of lytic reactivation with dox (0 hpi), inhibited the phosphorylation of the downstream mTOR target S6 but had little effect on 4E-BP1 phosphorylation (Fig. 2, lane 7). In contrast, treatment with the mTORC1/mTORC2 active-site inhibitor Torin completely inhibited S6 and 4E-BP1 phosphorylation for the duration of the 72-h time course (Fig. 2, lane 9). Treatment with rapamycin at the time of dox-induced lytic reactivation (0 hpi) modestly inhibited viral protein accumulation (Fig. 2, lane 7), whereas Torin potently inhibited the accumulation of viral proteins across all temporal classes (RTA, ORF45, K8.1, and ORF65) (Fig. 2, lane 9). Delaying treatment with Torin or rapamycin to 24 hpi yielded similar phosphorylation patterns for S6 and 4E-BP1 target proteins as at 0 hpi, suggesting that mTORC1 was required for phosphorylation of these canonical target proteins at the IE and E stages of lytic replication. However, delaying the delivery of Torin or rapamycin to 24 hpi allowed late viral proteins to accumulate to levels comparable to those of vehicle-treated controls over the subsequent 48 h (Fig. 2, lanes 8 and 10). These findings suggest that even though mTORC1 is active during KSHV lytic replication, it may be dispensable for the synthesis of viral proteins in middle and late stages of the lytic replication cycle.

### mTORC1 is dispensable for progression from early to late replication.

**FIG 3:**
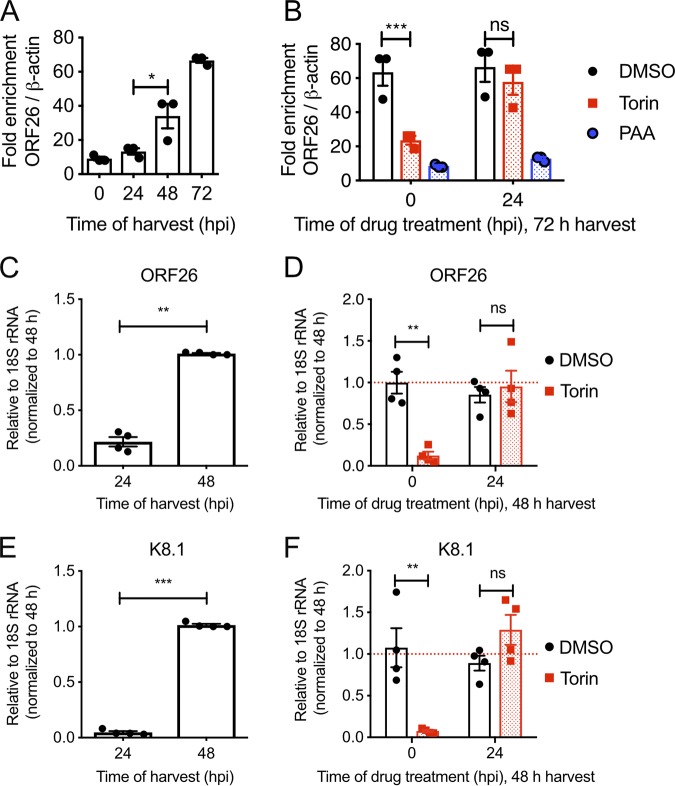
mTORC1 is dispensable for progression from early to late KSHV replication. (A) Viral genome replication. iSLK.219 cells were reactivated with 1 μg/ml doxycycline. DNA was extracted from iSLK.219 cells at the indicated times, and the relative proportion of KSHV to human genomes was determined by comparing the proportions of ORF26 and β-actin by qPCR (*n* = 3; means ± standard errors of the means [SEM]; statistical significance was determined by one-way analysis of variance [ANOVA]). (B) Cells were treated as indicated with DMSO, 250 nM Torin, or 500 μM PAA at either 0 or 24 hpi, and qPCR was performed to quantify KSHV intracellular genomes (*n* = 3; means ± SEM; statistical significance was determined by two-way ANOVA). (C to F) Quantification of viral late gene expression. (C and E) RNA was extracted at the indicated times from reactivated iSLK.219 cells, and RT-qPCR was performed for the late genes ORF26 (C) and K8.1 (E) (*n* = 4; means ± SEM; statistical significance was determined by one-way ANOVA). (D and F) iSLK.219 cells were treated with DMSO or 250 nM Torin at 0 or 24 hpi, and RNA was harvested at 48 hpi. RT-qPCR was performed for the late genes ORF26 (D) and K8.1 (F) (*n* = 4; means ± SEM; statistical significance was determined by two-way ANOVA).

Accumulation of KSHV late proteins in the presence of rapamycin and Torin (Fig. 2) suggests that mTORC1 activity could be dispensable for genome replication and progression through the lytic cycle. To test this directly, DNA was extracted from infected cells and processed for quantitative PCR (qPCR) amplification of the viral ORF26 gene and the human β-actin gene, which enabled quantitative monitoring of viral genome replication over time. Treatment of iSLK.219 cells with dox and harvesting of DNA at 0, 24, 48, and 72 hpi for qPCR revealed that viral genome replication began at some time between 24 and 48 hpi and increased further by 72 hpi (Fig. 3A). Treatment of control cells with PAA at either 0 hpi or 24 hpi completely blocked viral genome replication (Fig. 3B), confirming that genome replication occurs after the 24-h mark in this model. Coadministration of dox and Torin greatly diminished viral genome replication over the 72-h time course (Fig. 2B), whereas delaying Torin treatment to 24 hpi allowed efficient genome replication compared to that in mock-treated control cells. To directly monitor the accumulation of late mRNAs in this system, we harvested RNA from dox-treated iSLK.219 cells and amplified late ORF26 and K8.1 transcripts by reverse transcription-qPCR (RT-qPCR); we observed that these late transcripts did not accumulate until 48 hpi (Fig. 3C and E), which was after genome replication had occurred. Accumulation of these late transcripts was inhibited by coadministration of Torin with dox at 0 hpi (Fig. 3D and F), whereas administration of Torin at 24 hpi had no effect. These observations suggest that once the virus entered early replication, mTORC1 activity was dispensable for viral genome replication and late gene expression.

### mTORC1 activity is dispensable for production of infectious virions after KSHV reactivation.

**FIG 4:**
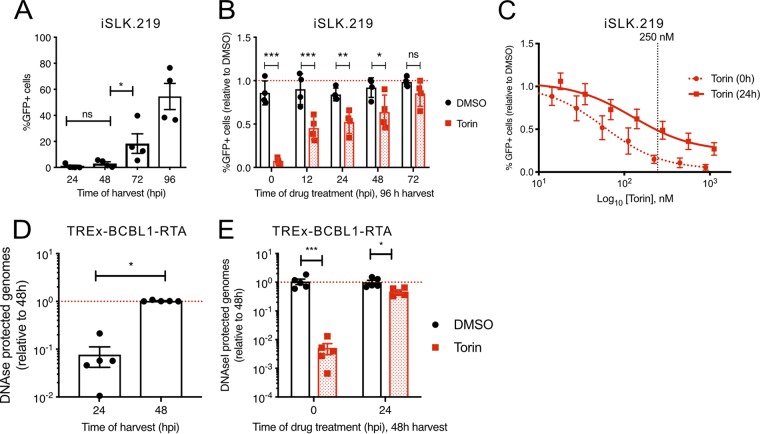
mTORC1 activity is dispensable for the production of infectious virions after KSHV reactivation. (A) iSLK.219 cells were treated with 1 μg/ml dox 1 day after seeding. The supernatant was harvested at the times indicated and frozen at −80°C until the titer was determined on naive 293A cells. GFP-positive (GFP^+^) cells from an arbitrary live-dead gate were recorded by flow cytometry (*n* = 4; means ± SEM; statistical significance was determined by one-way ANOVA). (B) Same as for panel A except that 250 nM Torin or DMSO was added to the cells at the time of doxycycline treatment (0 hpi) or at the times indicated. The supernatant was harvested at 96 hpi for titer determination as described above for panel A (*n* = 4; means ± SEM; statistical significance was determined by two-way ANOVA). (C) Same as for panel B except that iSLK.219 cells were treated with a range of Torin concentrations (16 nM to 1 μM) at 0 or 24 hpi (*n* = 5; means ± SEM). (D) TRex-BCBL1-RTA cells were reactivated from latency with 1 μg/ml dox. Cells and debris were removed by centrifugation, and the supernatant was treated with DNase I before harvesting virion DNA. DNase-protected viral genomes were quantified by qPCR using a luciferase plasmid as a recovery control (*n* = 5; means ± SEM; statistical significance was determined by Student’s *t* test). (E) Same as for panel D except that 250 nM Torin or DMSO was added at 0 or 24 hpi (*n* = 5; means ± SEM; statistical significance was determined by two-way ANOVA).

To determine whether mTOR inhibition affects virion production, supernatants from Torin-treated iSLK.219 cells or vehicle-treated control cells were collected and used to infect naive 293A cell monolayers. Because this virus constitutively expresses green fluorescent protein (GFP), infected 293A cells can be rapidly enumerated by flow cytometry. The first virions were produced by dox-treated iSLK.219 cells by as early as 48 hpi, and virion production increased further at 72 hpi and 96 hpi (Fig. 4A). Cells treated with Torin at 0 hpi produced very few infectious virions, but delaying Torin treatment to 12 hpi allowed approximately 50% virion production compared to the vehicle control (Fig. 4B). Delaying Torin treatment to 24, 48, and 72 hpi allowed for a steady increase in the release of infectious virions from these cells, such that treatment with Torin at 72 hpi (the final 24 h of the time course) produced nearly as many virions as the vehicle control. To determine the IC_50_ of Torin in this model, we treated iSLK.219 cells with a range of concentrations at the time of dox addition (*t* = 0 h) or 24 h later (*t* = 24 h) and harvested cell supernatants at 96 hpi as described above for determining the titer on 293A cells in the flow cytometry assay. When cells were treated with Torin at the time of reactivation, the IC_50_ in the virion production assay was 66 nM (Fig. 4C). In contrast, when Torin addition was delayed to 24 hpi, the IC_50_ was 121 nM. We corroborated these findings in TREx-BCBL1-RTA cells; these cells produce wild-type KSHV virions that lack a GFP transgene, so we were unable to determine the titer of virions using the flow cytometry assay. Instead, we used a qPCR assay to detect viral particles in the cell supernatant. Cell supernatants were collected at 0, 24, and 48 hpi and treated with DNase to digest any free KSHV DNA that was not protected by a capsid; capsids were subsequently disrupted in lysis buffer, and KSHV genomes were amplified by quantitative PCR. Using this assay, we measured few viral particles in the cell supernatant at 24 hpi, which increased by 48 hpi (Fig. 4D). Torin treatment at 0 hpi completely inhibited the release of DNase-protected genomes from TREx-BCBL1-RTA cells, but a delay of Torin treatment to 24 hpi allowed the assembly and release of approximately 50% as many viral particles as in vehicle control-treated cells (Fig. 4E). These results indicate that, similar to iSLK.219 cells, virion release from TRex-BCBL1-RTA cells was resistant to Torin treatment once virus replication had begun.

### mTORC1 inhibition does not affect release of VEGF-A or IL-6 during lytic replication.

**FIG 5:**
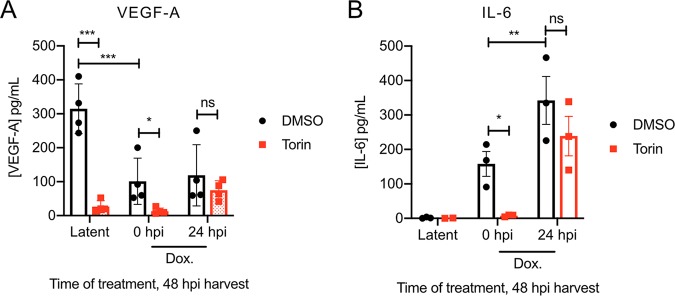
mTORC1 inhibition does not affect release of VEGF-A or IL-6 during lytic replication. (A) TREx-BCBL1-RTA cells were washed twice with PBS and seeded in fresh medium or medium supplemented with 1 μg/ml dox. A total of 250 nM Torin or DMSO was added at 0 or 24 h postseeding. Supernatants were harvested at 48 hpi, and cytokine concentrations of VEGF-A (A) and IL-6 (B) were quantified by an ELISA (*n* = 3 to 4; means ± SEM; statistical significance was determined by two-way ANOVA).

mTORC1 activity is normally required for efficient synthesis of the angiogenic growth factor VEGF-A and the proinflammatory cytokine interleukin-6 (IL-6), both of which have strong pathogenetic links to KSHV-associated malignancies that can be recapitulated in *in vitro* and *in vivo* models (17–19, 22). For example, PEL cell growth requires mTORC1-dependent expression of paracrine growth factors (19). We showed that mTORC1 inhibition with Torin inhibited VEGF-A production by latently infected TREx-BCBL1-RTA cells (Fig. 5A), confirming data from previous studies that employed the allosteric mTORC1 inhibitor rapamycin (19). VEGF-A accumulated during lytic replication but did not match levels produced by latently infected cells (Fig. 5A). No detectable IL-6 was produced by these cells during latency (Fig. 5B). Dox treatment in the presence of Torin at 0 hpi prevented the accumulation of IL-6, consistent with a failure of these cells to reactivate (Fig. 5B). The addition of Torin at 24 hpi did not significantly reduce levels of secreted VEGF-A or IL-6 collected from cell supernatants at 48 hpi (Fig. 5A and B). Combined with our above-described observations of sustained Torin-mediated dephosphorylation of 4E-BP1 during lytic replication (Fig. 2, lanes 9 and 10), these observations suggest that accumulation of VEGF-A and IL-6 during lytic replication may be less dependent on mTORC1 activity.

### mTORC1 promotes global protein synthesis during KSHV lytic replication.

**FIG 6:**
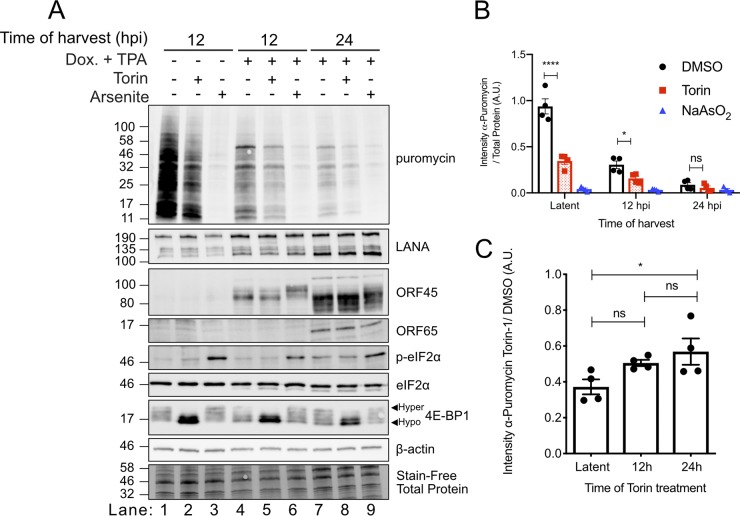
mTORC1 promotes global protein synthesis during KSHV lytic replication. (A) TREx-BCBL1-RTA cells were reactivated from latency with 1 μg/ml dox and 20 ng/ml TPA. Cells were treated with 250 nM Torin or DMSO for 2 h prior to harvest or with 500 μM sodium arsenite for 20 min prior to harvest. All cells were pulsed with 10 μg/ml puromycin 10 min prior to harvest in 2× Laemmli buffer. Viral replication was assessed by LANA (latent), ORF45 (early), and ORF65 (late) Western blotting. The incorporation of puromycin into nascent polypeptide chains indicates the rate or protein synthesis as detected by probing with an antipuromycin antibody. Sodium arsenite treatment phosphorylates Ser51-eIF2α, greatly limits translation, and acts a negative control for puromycin incorporation. (B) The puromycin intensity in panel A was quantified and compared to the total protein load as described in Materials and Methods (*n* = 4; means ± SEM; statistical significance was determined by two-way ANOVA). (C) Puromycin intensity of Torin-1 treatment from panel B relative to DMSO. Lane numbers correspond to lanes reading left to right in panel A (*n* = 4; means ± SEM; statistical significance was determined by one-way ANOVA). A.U., arbitrary units.

KSHV lytic replication features mTORC1 activation (12, 26, 35), but it is not known whether mTORC1 activity, and specifically mTORC1-mediated assembly of eIF4F, is required to support global protein synthesis during the lytic cycle. To address this directly, TREx-BCBL1-RTA cells were treated with dox and the phorbol ester 12-*O*-tetradecanoylphorbol-13-acetate (TPA) to ensure efficient lytic reactivation and harvested over a time course of infection (35). Puromycin was added to the cultures 10 min prior to harvest to measure new protein synthesis; puromycin resembles charged tyrosyl-tRNA and is incorporated at the carboxy terminus of nascent polypeptide chains, thereby preventing elongation. As the amount of puromycin incorporation is directly proportional to the quantity of mRNAs undergoing active translation, detection of puromycin by immunoblotting can be used as a proxy for global protein synthesis (44). We observed the highest level of puromycin incorporation into newly synthesized proteins in latently infected control cells (Fig. 6A, lane 1, and Fig. 6B); incorporation was reduced by more than 50% by 12 hpi (Fig. 6A, lane 4, and Fig. 6B) and approximately 90% by 24 hpi (Fig. 6A, lane 7, and Fig. 6B), consistent with previous reports of host shutoff during the KSHV lytic cycle by the viral endonuclease SOX (35, 45). In this assay, sodium arsenite served as a potent control for translation inhibition; treatment of cells with sodium arsenite for 10 min prior to the addition of puromycin (20 min prior to harvest) caused strong eIF2 phosphorylation and largely ablated puromycin incorporation (Fig. 6A, lanes 3, 6, and 9, and Fig. 6B). There was a comparable loss of global protein synthesis in all sodium arsenite-treated cells. Torin completely inhibited mTOR-mediated phosphorylation of 4E-BP1 in both latent and lytic cells (Fig. 6A, lanes 2, 5, and 8) and caused a significant loss of 50 to 60% of global protein synthesis compared to vehicle-treated cells (Fig. 6C) despite the diminished rate of protein accumulation during lytic replication. Together, these data suggest that mTORC1 activation is still required for protein synthesis even in the low-translation lytic environment.

### mTOR inhibition during latency and lytic replication disrupts eIF4F assembly.

**FIG 7:**
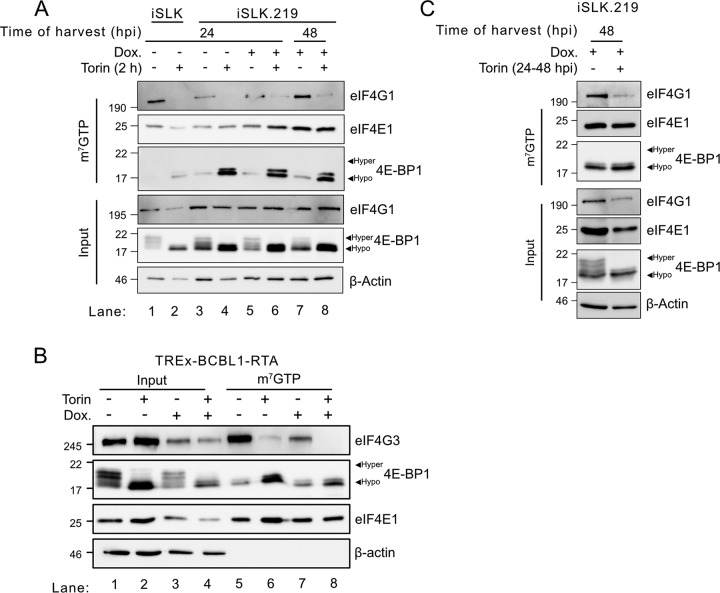
mTOR inhibition during KSHV latency and lytic replication disrupts eIF4F assembly. (A) Uninfected iSLK or iSLK.219 cells were reactivated with 1 μg/ml dox and treated with 250 nM Torin or DMSO for 2 h prior to harvest, as indicated. The cell lysate was incubated with m^7^GTP-agarose beads to isolate cap-binding protein complexes. To assess the integrity of eIF4F, input lysates and m^7^GTP pulldowns were probed for eIF4E1, 4E-BP1, and eIF4G1 or eIF4G3 by Western blotting. (B) m^7^GTP pulldown of latent TREx-BCBL1-RTA cells and cells reactivated with 1 μg/ml dox for 24 h. A total of 250 nM Torin or DMSO was added 2 h prior to harvest. (C) Cells were treated as described above for A except that iSLK.219 cells were treated with 250 nM Torin or DMSO for 24 h prior to harvest and Western blotting.

To further examine the role of mTORC1 activity in eIF4F regulation during lytic replication, we performed m^7^GTP-Sepharose bead pulldowns in native lysates from both latent and reactivated iSLK.219 and TREx-BCBL1-RTA cells and probed the assembly of canonical and alternative eIF4F constituent proteins in the presence or absence of Torin. eIF4G1 and eIF4E1 cap-binding proteins were coisolated in m^7^GTP-Sepharose bead pulldowns from latent and lytic iSLK.219 lysates (Fig. 7A), demonstrating the formation of canonical eIF4F complexes at all stages of viral replication. eIF4G1 was depleted from these complexes when cells were treated with Torin for 2 h prior to harvest, which was consistent with the reciprocal increase of hypophosphorylated 4E-BP1 detected in the pulldowns (Fig. 7A, lanes 4, 6, and 8). Similarly, Torin treatment of latent or 24-hpi TREx-BCBL1-RTA cells greatly reduced eIF4G3 coisolation, whereas 4E-BP1 recovery increased (Fig. 7B). Together, these data confirm that eIF4F can indeed be disrupted by mTORC1 inhibition during KSHV lytic replication.

To investigate the effects of long-term mTORC1 inhibition in this model, we treated lytic iSLK.219 cells with Torin at 24 hpi and harvested lysates for m^7^GTP-Sepharose bead pulldowns at 48 hpi. Similar to the 2-h Torin treatments shown in Fig. 7A, 24 h of sustained mTORC1 inhibition during lytic replication also impaired eIF4F assembly (Fig. 7C) while selectively inhibiting the accumulation of total eIF4G1. This indicates that there is no compensatory recovery of eIF4F during prolonged mTORC1 inhibition. These data suggest that, during the KSHV lytic cycle, eIF4F can assemble on mRNA cap structures and that direct mTORC1 inhibition by Torin treatment allows rapid dephosphorylation of 4E-BP1 and eIF4F disassembly. This suggests that eIF4F-independent translation initiation mechanisms may contribute to the Torin-resistant global protein synthesis that we observed during lytic infection (Fig. 6).

### Autophagy regulation during the lytic cycle is insensitive to mTORC1 signals.

**FIG 8:**
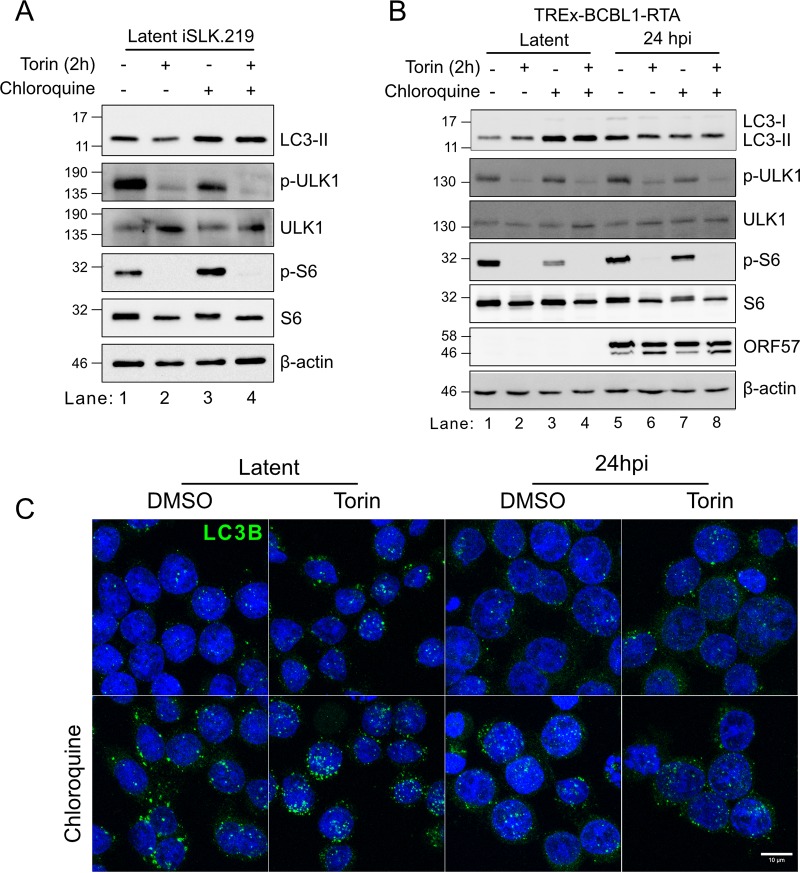
Autophagy regulation during the lytic cycle is insensitive to mTORC1 signals. (A) iSLK.219 cells were treated with DMSO, 250 nM Torin, or 50 μM CQ for 2 h prior to harvest. Autophagic flux was detected by accumulation of LC3-II by Western blotting. Phospho-ULK1 and phospho-S6 phosphorylations were probed to measure mTORC1 activity. (B) TREx-BCBL-RTA cells were reactivated with 1 μg/ml dox for 24 h before treatment with DMSO, 250 nM Torin, or 50 μM CQ. Either latent or 24-hpi cells were treated for 2 h prior to harvest and Western blotting as for panel A and for the early protein ORF57. (C) TREx-BCBL1-RTA cells were seeded onto poly-d-lysine-coated coverslips. The following day, cells were reactivated with 1 μg/ml dox. Latent and 24-hpi cells were treated with 250 nM Torin, DMSO, and chloroquine 24 h later, as indicated, for 2 h prior to harvest. Cells were washed with PBS, fixed with ice-cold methanol, and stained for LC3B. Nuclei were stained with DRAQ5. Maximum-intensity projections from z-stacks captured by confocal microscopy are shown.

mTORC1 inhibition by rapamycin, Torin, or starvation activates autophagy through loss of ULK1 phosphorylation (46, 47). Autophagy restricts herpesvirus replication, which the viruses counter by encoding autophagy antagonist proteins (37, 48). KSHV encodes at least three autophagy-restricting proteins, vBcl2, vFLIP, and K7/K-survivin, in addition to mTORC1-activating proteins (37–39). However, it is not known if these herpesvirus autophagy antagonist proteins are sufficient to limit autophagy in the absence of mTORC1 activity during lytic replication. Torin treatment of latent iSLK.219 cells led to dephosphorylation of the mTORC1 target proteins S6 and ULK1 (Fig. 8A). Using chloroquine (CQ) treatment to limit lysosomal degradation of LC3B-II, we observed limited autophagic flux during latency (Fig. 8A), which was unaffected by Torin treatment. Similar results were observed in latent TREx-BCBL1-RTA cells (Fig. 8B). In contrast, following 24 h of dox-mediated lytic replication, a 2-h treatment with Torin had little effect on LC3-II levels, despite inhibiting ULK1 phosphorylation (Fig. 8B, compare lanes 5 and 6), and CQ treatment revealed that Torin had no effect on the stymied autophagic flux in the pathway (Fig. 8B, compare lanes 7 and 8). We seeded TREx-BCBL1-RTA cells on cover glass with poly-d-lysine to observe autophagosomes by anti-LC3 immunofluorescence microscopy under the same conditions as the ones described in the legend of Fig. 8B. Autophagosomes can be observed during latency, and CQ treatment greatly enhances their abundance. While autophagosomes can be observed in lytic cells, treatment with Torin to inhibit mTORC1 or CQ to block autophagic flux did not appreciably alter LC3 staining (Fig. 8C). These results are consistent with observations of LC3-II levels in Fig. 8B. Together, these data suggest that autophagy was largely insensitive to perturbations in mTORC1 activity during KSHV lytic replication, which likely indicates a dominant role for viral autophagy-regulating proteins rather than viral mTORC1-activating proteins in governing autophagy during the lytic cycle, consistent with previous reports (37–39).

### Autophagy restricts KSHV replication.

**FIG 9:**
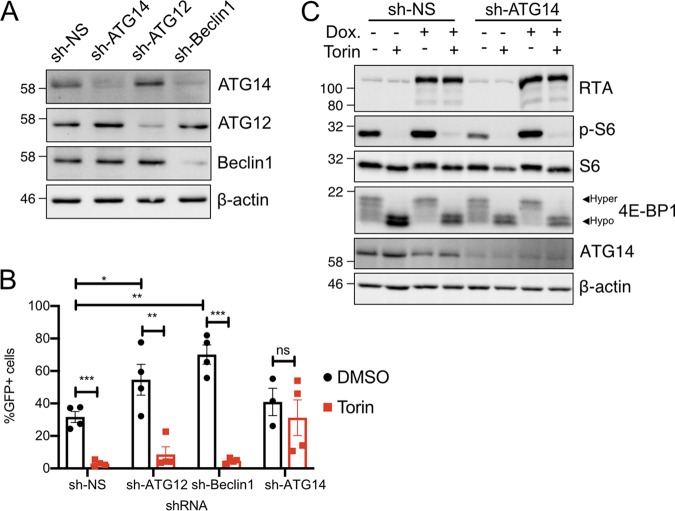
ATG14 is required for the inhibitory effects of Torin on reactivation and virion production. (A) shRNA knockdown of essential autophagy genes. iSLK.219 cells were transduced with lentiviruses encoding shRNAs for ATG14, ATG12, and Beclin1 or a nontargeting control shRNA (sh-NS). After selection with blasticidin, cell lysates were immunoblotted with the respective antibodies to confirm knockdown. (B) Production of virions from shRNA-transduced iSLK.219 cells reactivated with 1 μg/ml dox and treated with DMSO or 250 nM Torin. The supernatant was harvested at 96 hpi and used to infect a monolayer of 293A cells. Infected cells were enumerated as GFP^+^ events by flow cytometry (*n* = 3 to 4; means ± SEM; statistical significance was determined by two-way ANOVA). (C) iSLK.219 cells were transduced with an shRNA targeting ATG14 or a nontargeting construct and selected with blasticidin. Latent cells or cells reactivated with 1 μg/ml dox for 24 h were treated with 250 nM Torin or DMSO for 2 h prior to harvest and probed by Western blotting for mTORC1 activity with phospho-S6 and 4E-BP1. Virus reactivation was assessed by RTA expression.

Our data indicated that autophagy was insensitive to signals from mTORC1 during KSHV lytic replication. Still, a few autophagosomes could be observed during lytic replication, suggesting that the autophagy-limiting activities of viral proteins are not absolute. To test the role of autophagy in virus infection, we used short hairpin RNAs (shRNAs) to silence autophagy genes, ATG12, ATG14, or Beclin1, in iSLK.219 cells (Fig. 9A). We noted that Beclin1 silencing decreased the accumulation of ATG14, which was reported previously (49); we speculate that ATG14 stability may depend on complex formation with Beclin1. iSLK.219 cells bearing ATG12, ATG14, Beclin1, or nontargeting control shRNAs were treated with dox to induce reactivation from latency. We observed that Beclin1 or ATG12 silencing moderately increased virion production, as measured by collecting cell supernatants at 96 hpi and determining the titer of infectious virions on recipient 293A cell monolayers, whereas ATG14 silencing did not have a statistically significant effect compared to controls (Fig. 9B, black bars).

Because autophagic flux has been reported to control mTORC1 activity (50), we sought to determine whether viral regulation of autophagy could create the conditions for mTORC1-mediated control of reactivation from latency or mTORC1-insensitive viral protein synthesis later in the lytic cycle. As we observed previously, Torin treatment at the time of reactivation from latency dramatically decreased virion production, which was partially restored in cells deficient in ATG14 (Fig. 9B, red bars). Importantly, we did not observe any spontaneous reactivation from latency in the ATG14-deficient cells. Together, these observations suggest that Beclin1 and ATG14 may contribute to the restriction of KSHV replication by distinct mechanisms, with Beclin1 acting as a component of a constitutive inhibitory mechanism, whereas ATG14 is required to license the inhibitory effects of Torin on KSHV reactivation from latency. To ensure that ATG14 silencing was not affecting the ability of Torin to inhibit mTORC1, we treated latent ATG14-deficient iSLK.219 cells, or nontargeting shRNA control cells, with Torin for 2 h prior to lysis and Western blotting to measure S6 and 4E-BP1 phosphorylation. Lysates were also collected from cells that had been reactivated from latency with dox for 24 h before exposure to Torin for 2 h. We observed that ATG14 silencing had no effect on the phosphorylation of canonical mTORC1 target proteins during either KSHV latency or lytic replication, suggesting that ATG14 deficiency does not influence the uptake of the drug, normal mTORC1 targeting, or the activity of relevant phosphatases (Fig. 9C).

### mTORC1 inhibition limits RTA transgene accumulation.

**FIG 10:**
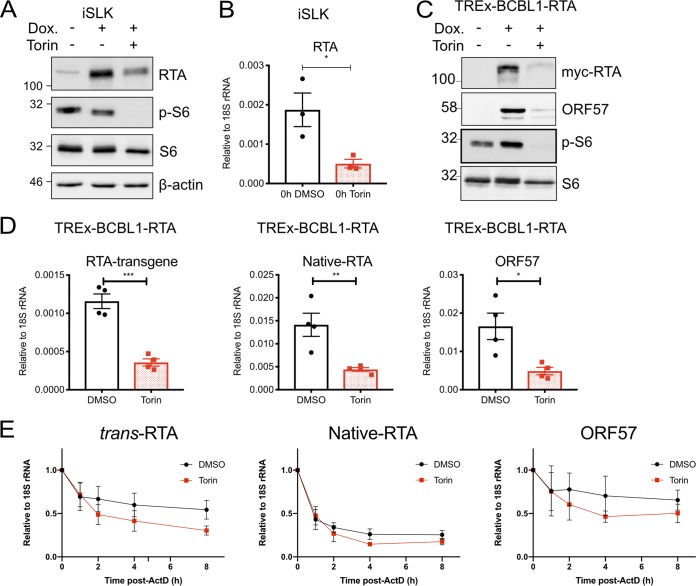
mTOR inhibition limits RTA accumulation. (A) Uninfected iSLK cells were treated with 1 μg/ml dox and 250 nM Torin or DMSO and probed for RTA transgene expression by Western blotting. (B) RT-qPCR analysis of uninfected iSLK cells treated as described above for panel A using primers targeting the coding DNA sequence (CDS) of RTA (*n* = 3; means ± SEM; statistical significance was determined by Student’s *t* test). (C) TREx-BCBL1-RTA cells were reactivated with 1 μg/ml dox and treated with 250 nM Torin or DMSO. RTA transgene expression was detected by probing for its myc epitope tag by Western blotting. (D) RT-qPCR analysis of TREx-BCBL1-RTA cells treated as described above for panel C using primers targeting the RTA transgene, native RTA, or the early gene ORF57 (*n* = 4; means ± SEM; statistical significance was determined by Student’s *t* tests). (E) TREx-BCBL1-RTA cells were reactivated with 1 μg/ml dox. Cells were treated with 250 nM Torin or DMSO and 10 μg/ml actinomycin D (ActD) at 16 hpi. RNA was harvested at 1, 2, 4, and 8 h posttreatment for RT-qPCR analysis as described above for panel D.

The addition of Torin at the time of dox-mediated reactivation from latency dramatically inhibited viral gene expression (Fig. 2, lane 9, and Fig. 3C and D), viral genome replication (Fig. 3B), and virion production (Fig. 4B) in the iSLK.219 cell model and diminished the production of viral particles by dox-treated TREx-BCBL1-RTA cells ∼100-fold (Fig. 4E). Because of these dramatic effects, we speculated that Torin treatment may inhibit lytic reactivation, either directly or indirectly. Lytic reactivation depends on the expression of RTA. While RTA is generally sufficient to promote reactivation from latency, the efficiency of reactivation varies between cell culture models and may benefit from additional signals. Rapamycin was previously shown to inhibit spontaneous, low-level, or chemically stimulated RTA expression in parental BCBL1 cells by preventing the accumulation of RTA mRNA (51), but it was not known whether the Tet-driven RTA transgene in iSLK.219 or TREx-BCBL1-RTA cells was subject to the same restriction. When iSLK.219 cells were treated with Torin at the time of dox addition and cell lysates were immunoblotted with anti-RTA antibody (Fig. 2, lane 9), we observed diminished levels of total RTA protein but were unable to determine whether this could be attributed to diminished levels of native RTA encoded by the viral genome, ectopic RTA encoded by the cell, or both. To determine whether accumulation of the cell-encoded RTA protein was sensitive to Torin, we treated uninfected iSLK cells with dox alone or coadministered dox and Torin for 24 h before harvesting lysates for Western blotting with an anti-RTA antibody. We observed that dox treatment alone dramatically increased RTA accumulation over the first 24 h, which was diminished but still detectable in the presence of Torin (Fig. 10A). Torin activity in this experiment was confirmed by immunoblotting for S6 phosphorylation; control cell lysates contained detectable phospho-S6, whereas Torin-treated cells had no detectable phospho-S6 despite little change in total S6 protein levels. RNA harvested in the same experiment was processed for RT-qPCR with gene-specific primers for RTA, which surprisingly revealed that Torin inhibited RTA mRNA accumulation in iSLK cells (Fig. 10B). We corroborated these observations in the TREx-BCBL1-RTA model, which encodes a dox-regulated myc epitope-tagged RTA protein. Treatment of TREx-BCBL1-RTA cells with dox alone caused accumulation of myc-RTA as determined by anti-myc Western blotting, which was dramatically reduced with Torin treatment (Fig. 10C). Again, Torin activity was confirmed by Western blotting for phospho-S6; lytic reactivation caused increased phospho-S6 levels, as observed in Fig. 2, which were reduced in Torin-treated cells. Using gene-specific primers, we determined that Torin inhibited the accumulation of the cell-encoded myc-RTA mRNA and the virally encoded RTA mRNA (Fig. 10D), which had the expected impact of diminishing levels of the early mRNA ORF57. To determine whether Torin affected RTA mRNA stability, TREx-BCBL1-RTA cells were treated with dox to induce lytic reactivation and RTA-dependent early gene expression for 16 h, followed by treatment with 250 nM Torin or the dimethyl sulfoxide (DMSO) control, along with 10 μg/ml actinomycin D to arrest *de novo* transcription. mRNA was harvested 0, 1, 2, 4, and 8 h after actinomycin D treatment and processed for RT-qPCR, which revealed that the stability of the RTA transgene mRNA (“*trans*-RTA”) was not significantly affected by Torin (Fig. 10E). Likewise, the stability of the native RTA mRNA transcribed from the viral genome was unaffected by Torin, even though it was more labile than the “*trans*-RTA.” The RTA-dependent early mRNA ORF57 was also tested, and it showed no significant alterations in mRNA stability in the presence of Torin. These data indicate that Torin inhibits the overall accumulation of RTA mRNA in both the iSLK and TREx-BCBL1-RTA cell models but does not accelerate RTA mRNA turnover.

### The mTORC1 requirement for reactivation from latency can be overcome by the histone deacetylase inhibitor sodium butyrate.

**FIG 11:**
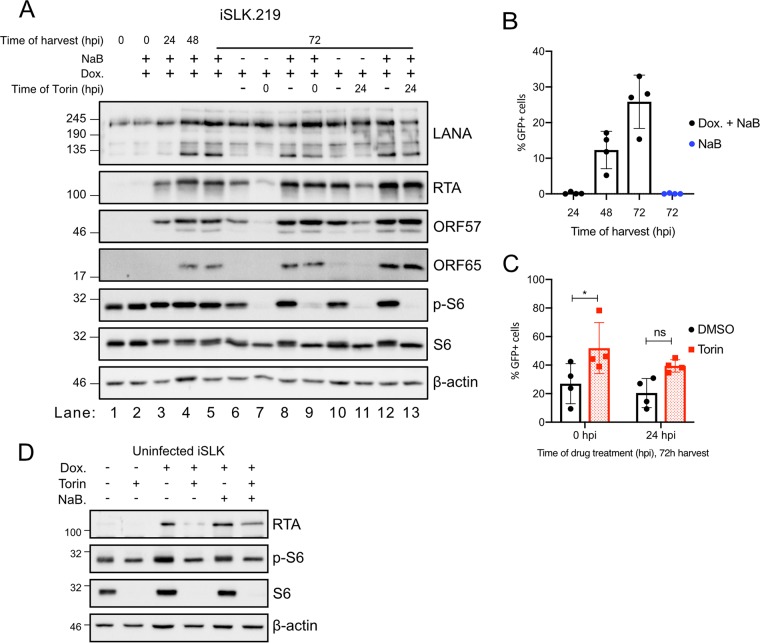
The mTORC1 requirement for reactivation from latency can be overcome by the histone deacetylase inhibitor sodium butyrate. (A) iSLK.219 cells reactivated with 1 μg/ml dox with or without 1 μM sodium butyrate (NaB) treatment were treated with 250 nM Torin or DMSO at either 0 or 24 hpi and harvested at the times indicated. Lysates were immunoblotted to detect the latent protein LANA, the immediate early protein RTA, the early protein ORF57, and the late protein ORF65. mTORC1 activity was determined by immunoblotting for phospho-S6. (B) Supernatants from iSLK.219 cells reactivated with 1 μg/ml dox and 1 mM NaB were used to infect a monolayer of 293A cells. Infected GFP^+^ 293A cells were enumerated by flow cytometry (*n* = 4; means ± SEM; statistical significance was determined by one-way ANOVA). (C) Same as for panel B except that 250 nM Torin or DMSO was added to the cells at the time of reactivation or at 24 hpi (*n* = 4; means ± SEM; statistical significance was determined by two-way ANOVA). (D) Uninfected iSLK cells were treated as indicated with dox or dox plus NaB. Cells were treated with 250 nM Torin or DMSO, and lysates were harvested 24 h later and probed for accumulation of the RTA transgene by Western blotting. mTORC1 activity was determined by immunoblotting for phospho-S6.

Reactivation of iSLK.219 cells is more efficient when dox treatment is supplemented with the histone deacetylase (HDAC) inhibitors sodium butyrate (NaB) and valproic acid (VPA) (52), which decondense chromatin on the viral episome, including at the RTA promoter (53, 54). NaB and VPA are sufficient to reactivate KSHV from latency in several cell lines, including BCBL1 cells, but neither is sufficient to stimulate lytic reactivation in SLK cells (4, 52). Lytic KSHV reactivation in iSLK.219 cells by coadministration of dox and NaB caused accumulation of RTA and the early protein ORF57 by 24 hpi (Fig. 11A, lane 3), whereas the late ORF65 capsid protein accumulated by 48 hpi (Fig. 11A, lane 4). The addition of Torin at the time of reactivation with dox alone dramatically inhibited the subsequent accumulation of KSHV lytic proteins over the next 72 h, including RTA, ORF57, and ORF65, while potently suppressing S6 phosphorylation (Fig. 11A, lane 7). In contrast, Torin treatment at the time of reactivation in the presence of dox and NaB had no effect on the accumulation of viral proteins (Fig. 11A, lane 9). Infectious virions could be detected from these reactivated cultures (Fig. 11B), and Torin treatment at the time of reactivation significantly enhanced virion yields (Fig. 11C). Importantly, NaB treatment did not interfere with Torin-mediated inhibition of mTORC1 activity (Fig. 11A, lanes 9 and 13). We found that Torin treatment at the time of dox addition prevented the accumulation of RTA transgene mRNA and protein in the iSLK and TREx-BCBL1-RTA cell lines (Fig. 10). Reactivation with dox and NaB partially rescued RTA transgene expression in the uninfected parental iSLK cell model (Fig. 11D). These findings indicate that HDAC inhibition relieves the requirement of mTORC1 activity for efficient reactivation from latency.

## DISCUSSION

The seminal discovery that the allosteric mTORC1 inhibitor rapamycin (sirolimus) caused regression of iatrogenic Kaposi’s sarcoma (KS) lesions suggested a central role for mTORC1 in KSHV biology (16). mTORC1 activity was subsequently shown to be required for the production of host proinflammatory cytokines and angiogenic factors in KS models (17–19, 22). In agreement with these findings, we observed that mTORC1 activity contributed to global protein synthesis and cytokine release from latently infected cells. mTORC1 also contributed to global protein synthesis during lytic replication, but once lytic replication had begun, the accumulation of two viral late proteins was largely resistant to rapamycin- or Torin-mediated mTORC1 inhibition and eIF4F disassembly. Accordingly, mTORC1 activity was largely dispensable for viral genome replication, late gene expression, and the release of infectious viral progeny.

During lytic replication, the SOX host shutoff protein causes widespread degradation of host and viral mRNAs that should reduce competition for eIF4F. However, we observed that eIF4F was still required for ∼50% to 60% of the total protein synthesis in lytic cells, which is similar to previous reports of the contribution of eIF4F to global protein synthesis under standard cell culture conditions of rich medium and normoxia (56). The residual eIF4F-independent protein synthesis in mTORC1-inhibited cells is handled by a collection of non-eIF4F translation initiation factors, such as N^6^-methyladenosine (m^6^A)-dependent translation initiation or eIF4E2-dependent translation during hypoxia (57, 58). Several studies have reported m^6^A modification of KSHV mRNAs, and m^6^A modification of RTA is required for proper splicing (59–61); however, for the most part, precise roles for m^6^A modifications and m^6^A-binding proteins in the biogenesis and fate of KSHV mRNAs remain to be elucidated and may vary depending on the cell type and different chemical stimuli of lytic reactivation (59). Our findings are similar to those in a previous report by Lenarcic and colleagues showing that the synthesis of HCMV proteins was increasingly resistant to mTORC1 inhibition as lytic replication progressed (62). While this study and our experiments do not explore the specific events beyond eIF4F assembly in the translation of viral mRNA, the accumulation of late gene products and infectious virions in the absence of eIF4F suggests that translation of some herpesvirus mRNAs can be initiated by non-eIF4F factors.

Our study extends the understanding of mTORC1-dependent reactivation from latency. Nichols et al. previously demonstrated that rapamycin had no effect on the synthesis of KSHV latent proteins in the BCBL1 PEL cell line but prevented the accumulation of the RTA lytic switch protein following reactivation with the HDAC inhibitor valproic acid (VPA) (51). In contrast, rapamycin prevented the accumulation of RTA transcripts in BCBL1 cells reactivated by treatment with the phorbol ester TPA or with cobalt chloride (CoCl_2_), which stabilizes the hypoxia-inducing factor 1α (HIF-1α) transcription factor. Arias et al. demonstrated that rapamycin had little effect on RTA expression in the TREx-BCBL1-RTA cell model (35), but here, using the potent mTOR active-site inhibitor Torin, we observed that mTORC1 activity is required for reactivation from latency in both the iSLK.219 and the TREx-BCBL1-RTA cell models. Furthermore, Torin inhibited the accumulation of the dox-inducible RTA transgene mRNA as well as the native RTA encoded by the viral genome in both cell models without affecting RTA mRNA stability. Combined with these previous studies, our findings suggest a model whereby RTA transcription depends on mTORC1 activity. This model is supported by our observation that the HDAC inhibitor sodium butyrate rescued RTA expression and dox-induced lytic reactivation in Torin-treated TREx-BCBL1-RTA cells. We do not know how mTORC1 activity supports RTA transcription, but it is well known to exert epigenetic and transcriptional effects in a variety of models (reviewed in reference 63). For example, in the fission yeast Schizosaccharomyces pombe, TOR1 and TOR2 control nutrient-responsive gene transcription by controlling the activity of the Spt‐Ada‐Gcn5 acetyltransferase (SAGA) complex, a megadalton transcriptional coactivator complex that features histone acetyltransferase (HAT) activity (64). SAGA is conserved between yeast and humans, but SAGA regulation by mTORC1/2 in humans or other mammals has not yet been reported. We speculate that RTA transcription may be especially reliant on histone acetylation in these cell models, which could depend on either intact mTORC1 activity or exogenous HDAC inhibitors like VPA or sodium butyrate.

mTORC1 normally inhibits autophagy in nutrient-rich environments by phosphorylating and inactivating autophagy-initiating kinases like ULK1, ULK2, and ATG13; nutrient deprivation causes mTORC1 inactivation and derepression of these kinases and initiation of autophagy (46, 47). We observed that amino acid withdrawal inactivated mTORC1 as expected in latently infected cells, but during lytic replication, mTORC1 kinase targets were constitutively activated and insensitive to AA withdrawal. We speculate that the viral proteins K1 and vGPCR may indirectly stimulate mTORC1 activity despite amino acid withdrawal in our system, consistent with previous reports (42, 74). However, it is also possible that an as-yet-unidentified viral protein may aid in the recruitment of mTORC1 complexes to the surface of lysosomes to hold them in an active state.

Autophagy is thought to restrict herpesvirus replication, which the viruses counter by encoding autophagy antagonist proteins (37, 48). KSHV encodes at least three autophagy-restricting proteins, vBcl2, vFLIP, and K7, in addition to the above-mentioned mTORC1-activating proteins (37–39). However, it is not yet known if these herpesvirus autophagy antagonist proteins are sufficient to limit autophagy in the absence of mTORC1 activity during lytic replication. We observed that autophagy was largely insensitive to perturbations in mTORC1 activity during KSHV lytic replication in the iSLK.219 and TREx-BCBL1-RTA cell models, which likely indicates a dominant role for viral autophagy-regulating proteins rather than viral mTORC1-activating proteins in governing autophagy during the lytic cycle.

In the course of these studies, we discovered that ATG14 is required for mTORC1 dependence of KSHV reactivation from latency, even though ATG14 silencing did not perturb mTORC1 activity. Several essential autophagy proteins have been shown to play additional roles in other cellular processes (65, 66). Similarly, viruses can usurp specific components of the autophagy pathway during different phases of replication (67). Kim and colleagues demonstrated a nonautophagy role for ATG14 in regulating endosome maturation by binding to the SNARE-associated protein Snapin and influencing endocytic trafficking (68). ATG14 silencing delayed endosomal maturation, as evidenced by slowed kinetics of internalized receptor degradation. In contrast, ATG14 silencing had no effect on autophagic degradation. We speculate that dysregulation of endocytic trafficking in ATG14-deficient cells may affect the ability of mTORC1 to influence the latent-to-lytic switch.

Altogether, our studies lead us to conclude that while mTORC1 is active during KSHV lytic replication and is likely important for the viral replication cycle, and certainly important for pathogenesis, it may not be strictly required for the translation of viral mRNA. This suggests that viral transcripts may access alternative translation initiation machinery during lytic replication. Whether this machinery is accessed from the large repertoire of host translation factors, or is encoded by the viral genome, remains to be determined.

## MATERIALS AND METHODS

### Cell lines.

293A cells were obtained from Thermo Fisher Scientific (catalog number R70507). iSLK and iSLK.219 cells (52) were a kind gift from Don Ganem. iSLK cells are a subclone of Caki-1 kidney epithelial cells that have been modified to carry the KSHV immediate early protein RTA under the control of a doxycycline-regulated promoter (52, 69). iSLK.219 cells are a subclone of iSLK cells latently infected with recombinant rKSHV.219 virus (52, 71). These cells were cultured in Dulbecco’s modified Eagle’s medium (DMEM) with 10% (vol/vol) heat-inactivated FBS, 100 IU of penicillin, and 100 IU of streptomycin. TREx-BCBL1-RTA cells (72), a kind gift from Jae Jung, were cultured in RPMI 1640 supplemented with 10% (vol/vol) heat-inactivated FBS, 55 μM β-mercaptoethanol, 100 IU of penicillin, and 100 IU of streptomycin. Amino acid starvation experiments with TREx-BCBL1-RTA cells were done using AA-free RPMI (Wisent) and dialyzed, heat-inactivated FBS (Thermo Fisher). Like iSLK cells, TREx-BCBL1-RTA cells express RTA from a doxycycline-regulated promoter. All cells were maintained at 37°C in a 5% carbon dioxide atmosphere. iSLK.219 cells were cultured in the presence of 10 μg/ml of puromycin (Thermo Fisher) to maintain the copy number of the episomal rKSHV.219 genomes (52, 71). Puromycin was not included in the medium of cells seeded for experiments. iSLK.219 cells were diluted to a density of 10^5^ cells/ml for seeding in all experiments. To induce *RTA* transgene expression in iSLK.219 cells, on the day following seeding, medium was refreshed and supplemented with 1 μg/ml doxycycline (Sigma). TREx-BCBL1-RTA cells were resuspended at a density of 2.5 × 10^5^ cells/ml and supplemented with 1 μg/ml doxycycline, with or without 20 ng/ml the phorbol ester 12-*O*-tetradecanoylphorbol-13-acetate (TPA), to induce *RTA* transgene expression.

### Chemicals.

Torin-1, referred to as “Torin” here (Toronto Research Chemicals) (24), and rapamycin (Sigma) were resuspended in DMSO, which was used as a vehicle control at 0.1% (vol/vol) in all experiments. Unless otherwise stated, both rapamycin and Torin were used at a concentration of 250 nM. Phosphonoacetic acid (PAA; Sigma) was used at 500 μM, chloroquine diphosphate (CQ; Sigma) was used at 50 μM, sodium butyrate (NaB; Sigma) was used at 1 mM, and sodium arsenite (Sigma) was used at 500 μM. Actinomycin D (Sigma) was resuspended in DMSO and used at 10 μM.

### Western blotting.

TREx-BCBL1-RTA cells are suspension cells. To harvest these cells, medium was collected, and cells were pelleted by centrifugation at 1,500 × *g* for 5 min. Cells were washed with ice-cold phosphate-buffered saline (PBS) and pelleted again. The supernatant was then removed, and the pellet was lysed with 2× Laemmli buffer (4% [wt/vol] sodium dodecyl sulfate [SDS], 20% [vol/vol] glycerol, 120 mM Tris-HCl [pH 6.8]). Adherent cells were first washed once with ice-cold PBS and then lysed in 2× Laemmli buffer. When harvesting lysates from lytic cell populations, nonadherent cells were collected and combined with adherent cells that had been removed from the dish with a rubber scraper in ice-cold PBS; adherent and nonadherent cells were isolated by centrifugation as described above. Cell pellets were lysed with 2× Laemmli buffer. DNA was sheared by repeated pipetting with a fine-gauge needle, and a sample of the lysate was reserved for protein quantification. A total of 100 mM dithiothreitol (DTT) was added to each sample prior to boiling at 95°C for 5 min. Samples were stored at −20°C prior to analysis.

The protein concentration in whole-cell lysates was quantified with a DC protein assay (Bio-Rad), and equal quantities of protein were loaded for SDS-PAGE and transferred to polyvinylidene difluoride (PVDF) membranes (Bio-Rad) using a Trans-Blot Turbo transfer apparatus (Bio-Rad). For proteins isolated from pulldowns or precipitated from polysome fractions, equal volumes of the lysate were loaded. Membranes were blocked with 5% (wt/vol) skim milk in TBS-T (Tris-buffered saline, 0.1% [vol/vol] Tween) or 5% (wt/vol) bovine serum albumin (BSA) in TBS-T and probed at 4°C overnight with primary antibodies as indicated. 4E-BP1 (catalog number 9644), β-actin–horseradish peroxidase (HRP) (catalog number 5125), ATG12 (catalog number 4180), ATG14 (catalog number 5504), eIF2α (catalog number 9722), phospho-Ser51-eIF2α (catalog number 3597), eIF4E1 (catalog number 2067), eIF4G1 (catalog number 2858), myc (catalog number 2276), Rps6 (S6) (catalog number 2217), phospho-Ser235/6-Rps6 (catalog number 4858), phospho-Ser757-ULK1 (catalog number 14202), and ULK1 (catalog number 8054) antibodies were purchased from Cell Signaling Technologies (CST). ORF57 (catalog number sc-135746) and Beclin1 antibodies were purchased from Santa Cruz Biotechnologies. ORF45 (catalog number MA5-14769) antibody was purchased from Thermo Fisher. K8.1 antibody was purchased from ABI (catalog number 13-212-100). LC3B antibody was purchased from either CST (catalog number 2755) or Novus (catalog number NBP2-19337). Antiserum for LANA was a gift from Don Ganem, and antiserum for RTA was a gift from David Lukac. A mouse monoclonal antibody for ORF65 was a gift from S.-J. Gao. Primary antibody binding was detected with horseradish-peroxidase conjugated anti-mouse (catalog number 7076) and anti-rabbit (catalog number 7074) secondary antibodies purchased from CST. Blots were developed with Clarity-ECL chemiluminescence reagent (Bio-Rad) and imaged on a Bio-Rad ChemiDoc-Touch system. Molecular weight markers (in kilodaltons) are noted on the left of cropped blot images.

### m^7^GTP pulldown.

A total of 2 × 10^6^ iSLK cells or iSLK.219 cells or 5 × 10^6^ TRex-BCBL1-RTA cells were used for each pulldown. After treatment, cells were washed twice with ice-cold PBS and scraped for harvesting. The cells were centrifuged for 5 min at 1,000 × *g*, and the pellet was lysed, on ice for 10 min, in a solution containing 20 mM Tris-HCl, 150 mM NaCl, and 0.5% (vol/vol) NP-40 with protease and phosphatase inhibitors. The lysate was centrifuged for 5 min at 10,000 × *g*, and the supernatant was precleared with a 50-μl settled volume of unconjugated agarose beads (Jena Biosciences) by incubation, with end-over-end rotation, for 10 min at 4°C. The beads were pelleted by centrifugation for 30 s at 500 × *g*, and 5% of the supernatant was removed as the input control. The remaining supernatant was incubated with m^7^GTP-agarose beads (Jena Biosciences) for 4 to 6 h at 4°C, with agitation. The beads were washed four times with lysis buffer. To harvest bound protein, the beads were then resuspended in 1× Laemmli buffer with 100 mM DTT and boiled at 55°C for 10 min. Equal volumes of lysates were analyzed by Western blotting as described above.

### Viral genome amplification and RT-qPCR.

DNA was harvested from the cells using a QIAamp DNA minikit (Qiagen) according to the manufacturer’s instructions. qPCR was performed using oligonucleotide primer sets for the ORF26 and β-actin genes and Go*Taq* polymerase enzyme (Promega). The KSHV genome copy number is represented as the fold increase for ORF26 compared to β-actin. For RT-qPCR, total RNA was extracted using RNeasy extraction (Qiagen). For RNA stability experiments, cells were treated with 10 μg/ml of actinomycin D to inhibit transcription, and total RNA was harvested with RNeasy 1, 2, 4, and 8 h after actinomycin D treatment. qPCR was performed using Go*Taq* polymerase as described above. cDNA was generated using MaximaH reverse transcriptase enzyme (Thermo Fisher), using random hexamers for priming, according to the manufacturer’s instructions. Both adherent and nonadherent cells were harvested in each sample. Transcripts are normalized to the abundance of 18S rRNA using the ΔΔ*C_T_* method. Primer sequences are listed in Table 1. An annealing temperature of 60°C was used for all primers.

**TABLE 1:** Oligonucleotide sequences

qPCR target	Primer sequence (5′–3′)	
	Forward	Reverse
18S	TTCGAACGTCTGCCCTATCAA	GATGTGGTAGCCGTTTCTCAGG
*luc2*	TTCGGCAACCAGATCATCCC	TGCGCAAGAATAGCTCCTCC
β-Actin	CTTCCAGCAGATGTGGATCA	AAAGCCATGCCAATCTCATC
ORF26	CAGTTGAGCGTCCCAGATGA	GGAATACCAACAGGAGGCCG
ORF57	TCCAGTTTTGCTCCCCACTG	TTCTGCCGTATTGTAGGCGG
K8.1	AGATACGTCTGCCTCTGGGT	AAAGTCACGTGGGAGGTCAC
RTA (CDS)	GATTACTGCGACAACGGTGC	TCTGCGACAAAACATGCAGC
Native RTA	CCGAGACTGAAGTGTTCGCA	AACGGAGGAAATACCACCCC
*trans*-RTA	ACTGTACCAGCTGCACCAAT	GGGAGGGGCAAACAACAGAT

### KSHV infection and titer determination by flow cytometry.

rKSHV.219 contains a green fluorescent protein (GFP) cassette controlled by a constitutive EF1α promoter, allowing infected cells to be identified by green fluorescence (71). The supernatant was harvested from iSLK.219 cells as indicated and stored at −80°C until titer determination. One day prior to titer determination, 2.5 × 10^5^ 293A cells were plated into each well of a 12-well plate. The thawed inoculum was mixed by inversion and centrifuged at 5,000 × *g* for 5 min to pellet debris. Cells were then infected with the diluted inoculum and centrifuged at 800 × *g* for 90 min (73). Immediately after spinoculation, the inoculum was removed, the cells were washed once with PBS, fresh medium was added, and the cells were returned to an incubator. The day following infection, 293A cells were detached with trypsin, washed once in cold PBS, and resuspended in PBS with 1% (vol/vol) paraformaldehyde (PFA). Flow cytometry was performed using a FACSCalibur instrument (BD Biosciences), using arbitrary forward- and side-scatter gates. The number of GFP-expressing cells among a batch of 10,000 cells was recorded for each sample. Data were analyzed using Flowing software version 2.5 (Perttu Terho, Turku Centre for Biotechnology, Finland [www.flowingsoftware.com]).

### DNase-protected viral genome qPCR.

At the time of harvest, the cells and debris were pelleted for 5 min at 5,000 × *g*. A total of 180 μl of the supernatant was mixed with 20 μl of 3 mg/ml DNase I (Sigma) and incubated for 30 min at 37°C. DNA was then extracted from the supernatant using a DNeasy blood and tissue minikit (Qiagen) according to the manufacturer’s instructions, with the following modifications: lysis buffer AL was spiked with 5 μg/sample of salmon sperm DNA (Invitrogen) and 1 ng of the luciferase (*luc2*)-containing plasmid pGL4.26 (Clontech). qPCR was performed with primers for the ORF26 gene to detect viral genomes and the *luc* carrier plasmid using Go*Taq* polymerase enzyme (Promega). The ORF26 quantity was normalized to *luc* using the ΔΔ*C_T_* method.

### Enzyme-linked immunosorbent assay.

The concentration of VEGF-A in the medium was measured using a human VEGF DuoSet enzyme-linked immunosorbent assay (ELISA) kit according to the manufacturer’s instructions (R&D Systems). IL-6 was detected using an in-house protocol using capture antibody (catalog number MQ2-13A5; Thermo Fisher) (1:1,000 dilution), detection antibody (catalog number MQ2-39C3; Thermo Fisher) (1:500 dilution), and a recombinant IL-6 standard (catalog number 14-8069-62; Thermo Fisher). Before seeding, cells were washed in PBS to remove any residual cytokines. The absorbance was measured on a plate reader at 450 nm with 570-nm correction subtracted. The cytokine quantity was calculated using a standard curve with Prism8 (GraphPad).

### Puromycin incorporation assay.

TREx-BCBL1-RTA cells were reactivated with 20 ng/ml of TPA and 1 μg/ml doxycycline. Cells were treated with 10 μg/ml puromycin for 10 min prior to harvest in 2× Laemmli buffer. Puromycin incorporation into nascent proteins was determined by Western blotting using an antipuromycin antibody (catalog number MABE343; EMD Millipore) (44). Western blot assays were performed using 4 to 15% Mini-Protean TGX stain-free gradient gels (Bio-Rad) according to the manufacturer’s instructions. For quantitative analysis, the total signal in the antipuromycin Western blot was normalized to the stain-free protein loading control.

### Immunofluorescence.

Glass coverslips (number 1.5; Zeiss) were coated with poly-d-lysine (Sigma) in PBS for 30 min at room temperature (RT) and then washed with PBS before seeding 2.5 × 10^5^ TREx-BCBL1-RTA cells. Cells were left to adhere overnight. After treatment, cells were washed once with PBS and fixed with ice-cold methanol. Cells were stored at −20°C in methanol before processing. The fixative was removed, and cells were washed three times for 5 min in PBS before cells were blocked in blocking buffer (1% [vol/vol] heat-inactivated human serum [Sigma] and 0.1% [vol/vol] Triton X-100 in PBS) for 1 h at RT with gentle agitation. Coverslips were stained with a 1:400 dilution of rabbit anti-LC3B (catalog number 2775; CST) in blocking buffer overnight at 4°C. Coverslips were washed three times with PBS for 5 min with gentle agitation before staining with a 1:1,000 dilution of donkey anti-rabbit Alexa 555 secondary antibody (Thermo Fisher) for 1 h at RT. Coverslips were briefly washed with PBS, incubated with 1 μM DRAQ5 (catalog number 4084; CST) in PBS for 5 min, briefly rinsed with PBS, and then mounted with ProLong gold antifade reagent (Thermo Fisher). Slides were imaged on an LSM510 confocal microscope (Zeiss) with a 63× objective at 1.4× zoom using Zen software. Maximum-intensity projections from z-stacks are depicted. Contrast and pseudocolor were adjusted using Fiji (40), and images were assembled into figures using Affinity Designer for macOS (Serif).

### shRNA gene silencing.

shRNA was transduced using pLKO lentiviral transduction. Lentiviruses were generated in 293T cells by using polyethylenimine Max (catalog number 24765; Polysciences) to cotransfect the pLKO cassette, with the packaging plasmids pMD2.G (a gift from Didier Trono; Addgene plasmid 12259) and psPAX2 (a gift from Didier Trono; Addgene plasmid 12260). At 48 h posttransfection, the supernatant was harvested, filtered at 0.45 μm, and frozen in aliquots at −80°C. iSLK.219 cells were transduced with lentivirus overnight in the presence of 4 μg/ml Polybrene (hexadimethrine bromide; Sigma). The following day, 20 μg/ml blasticidin (Thermo Fisher) was added to the cultures, and cells were incubated for a further 2 days for selection before passage or seeding for experiments. Experiments were conducted in antibiotic-free medium. Transduced cells were freshly generated for every experiment and not maintained in culture for long periods. shRNA sequences for *Beclin1*, *ATG12*, and *ATG14* were selected from the RNAi Consortium (clone IDs: sh-Beclin1, TRCN0000033549; sh-ATG12, TRCN0000007393; sh-ATG14, TRCN0000142647). cDNA oligonucleotides were annealed and ligated between the AgeI and EcoRI sites of pLKO-blast (a gift from Keith Mostov; Addgene plasmid 26655) according to the RNAi Consortium shRNA cloning protocol (https://portals.broadinstitute.org/gpp/public/resources/protocols). A nontargeting shRNA sequence, pLKO-NS-BSD (a gift from Keith Mostov [41]; Addgene plasmid 26701), was used as a control in all experiments.

### Statistical analysis.

All values were imported into Excel (Microsoft) for tabulation and normalization. All qPCR calculations were conducted in Excel. Values were then imported into Prism8 (GraphPad) for statistical analysis and graphing. *P* values are represented in the figures (*, *P* < 0.05; **, *P* < 0.01; ***, *P* < 0.001; ns, nonsignificant).
